# The contribution of the Medicines Use Review (MUR) consultation to counseling practice in community pharmacies^☆^

**DOI:** 10.1016/j.pec.2011.05.007

**Published:** 2011-06

**Authors:** Asam Latif, Kristian Pollock, Helen F. Boardman

**Affiliations:** a Division of Social Research in Medicines and Health, School of Pharmacy, University of Nottingham, Nottingham, United Kingdom; b School of Nursing, Midwifery, and Physiotherapy, University of Nottingham, Nottingham, United Kingdom

**Keywords:** Counseling, Medicines Use Reviews, Patient centred, Patient–pharmacist communication, Pharmacy practice

## Abstract

**Objective:**

To understand the contribution of the Medicines Use Review consultation to counseling practice in community pharmacies.

**Methods:**

Qualitative study involving ten weeks of observations in two community pharmacies and interviews with patients and pharmacy staff.

**Results:**

‘Traditional’ counseling on prescription medicines involved the unilateral transfer of information from pharmacist to patient. Over-the-counter discussions were initiated by patients and offered more scope for patient participation. The recently introduced MUR service offers new opportunities for pharmacists’ role development in counseling patients about their medicines use. However, the study findings revealed that MUR consultations were brief encounters dominated by closed questions, enabling quick and easy completion of the MUR form. Interactions resembled counseling when handing out prescription medicines. Patients rarely asked questions and indeterminate issues were often circumvented by the pharmacist when they did. MURs did little to increase patients’ knowledge and rarely affected medicine use, although some felt reassured about their medicines. Pragmatic constraints of workload and pharmacy organisation undermined pharmacists’ capacity to implement the MUR service effectively.

**Conclusion:**

Pharmacists failed to fully realise the opportunity offered by MURs being constrained by situational pressures.

**Practice implications:**

Pharmacist consultation skills need to be reviewed if MURs are to realise their intended aims.

## 1 Introduction

Reforms to the United Kingdom (UK) community pharmacy contract in England and Wales in 2005 sought to address pharmacy's heavy reliance on dispensing prescriptions to generate income by moving to reward patient-centred services [1,2]. The ‘Medicines Use Review and Prescription Intervention’ (MUR) service is one of a number of policy initiatives which seek to help people better manage their medicines as well as reduce the cost of wasted and inappropriate use of medicines [1,3,4]. MURs involve pharmacists undertaking a private consultation with the patient and aim to improve ‘*knowledge, concordance and use of medicines*’ [3]. The MUR involves completion of a national standard form. Information that the pharmacist is expected to elicit from the patient in order to complete the form includes whether they use the medicine as prescribed, whether they know the medicine's purpose, if the formulation is appropriate and reported side effects. The format of the form is tick-box allowing a yes/no response to questions [3]. Patients are eligible for the service if they are taking two or more medicines and have been receiving pharmaceutical services from the pharmacy for at least three months. Pharmacies are entitled to claim £28 reimbursement from the NHS for each MUR performed with an annual maximum of 400. In England, 1.7 million MURs were conducted in the 2009–2010 financial year costing a total of £47.7m [5]. Pharmacists are required to include, in their discussion, both prescribed and over-the-counter (OTC) use of medicines within the MUR. The MUR initiative is in tune with other UK health policy commitments such as patient choice [6,7], re-shaping care around the patient [8] and greater involvement of patients in their medicines management [9]. Similar medication review schemes are provided in Australia [10], United States [11] and New Zealand [12]. Formalising the pharmacist's counseling role by providing payment, represents a means to enhance professional status. Greater emphasis on patient contact and counseling has been advocated by professional bodies and those wishing to reprofessionalise pharmacists’ activities [13–15].

Most patient–pharmacist interactions still occur on the ‘shop-floor’ when the pharmacist supplies dispensed medicines to patients. This encounter, described in the UK as ‘counseling’, typically seeks to ensure that the directions on the labels of dispensed products are understood [16]. Variations have been reported in how pharmacists counsel on prescribed medicines; for example, information on directions, medicine name, and indications for use were given more frequently than information on side effects, cautions and interactions [17,18]. However, the community pharmacist's consultative role remains undeveloped and the concept of patient counseling ill defined [19]. Shah and Chewning [20] found that the definition of patient counseling varied across studies with half conceptualising patient–pharmacist communication as information provision. The predominantly information-based focus of patient–pharmacist interaction means that counseling in the pharmacy context carries a somewhat different meaning to that in other settings, such as psychotherapy, where there is a process of subjective scrutiny and greater engagement with, and contribution from, the client [19,21].

Another patient–pharmacist interaction relates to OTC sales of medicines. These are initiated by the patient and involve the offer of a professional opinion about a course of action, whist allowing the final decision to lie with the patient [22]. This level of indeterminacy requires negotiation and contrasts with counseling offered on dispensed medicines where patients’ information needs may be assumed to have been addressed by the General Practitioner (GP) [21]. Although OTC interactions potentially offer more scope for the pharmacist to explore patient perspectives and concerns, they are usually problem-specific and have attracted criticism as lost opportunities to discuss wider health issues [21,23].

MURs present an opportunity for pharmacists to extend their currently limited counseling role in wider discussions of patient beliefs and concerns about their medicines. This paper aims to contextualise and better understand MUR consultations as they occur in the ‘real world’ setting of pharmacy practice and explores what they may additionally offer over ‘traditional’ patient–pharmacist interactions for prescription and OTC medicines.

## 2 Methods

Following approval from a local NHS Research Ethics Committee, two pharmacies, a multiple and an independent were recruited purposefully via personal contacts. Consent was obtained from the pharmacists and support-staff for five weeks of observations in each pharmacy. One-week placements over a 12-month period between November 2008 and October 2009 allowed the data collection and analysis phases to proceed simultaneously. Pharmacies were requested to display posters within the pharmacy to promote patient awareness of the study.

Observation notes were made (by AL) of all pharmacy activities, the working environment, staff–patient conversations and all activities relating to MURs. Ethnographically orientated unstructured observation methods were used which involved detailed observation of behaviour and talk to better understand the social setting in which people function in their natural environment. AL recorded all aspects of the phenomenon and the context in which they occurred that seemed relevant to the situation being studied. This produced rich qualitative data in order to uncover behaviours or patterns of which the participants themselves may not have been aware [24]. The triangulation of direct observation (researcher's accounts) with accounts provided by respondents in interviews provided a powerful means of understanding the complexity of respondents’ views, how these may shift contextually, the situational pressures which underlie them, and the resulting difference in what people ‘say’ and what they ‘do’ [25].

All pharmacists and support-staff were requested to identify and invite patients for MURs as per normal practice and to introduce the research to all those who accepted the offer of an MUR. All such patients agreed to be included in the research and for their MUR to be observed, at which point AL was introduced and explained what was involved. Thus, AL was able to observe nearly all MURs taking place during the period of fieldwork while he was undertaking observation in the pharmacies. All extracts from the observation notes that are presented in this paper are taken from detailed notes written up after each observation, rather than verbatim quotes. Pseudonyms have been used in quoted extracts to maintain respondents’ anonymity.

After the MUR patients were invited to take part in an interview about their experience. A topic guide was developed and tailored to the specific details and context of the MUR which preceded it (Box 1). The topic guide was used to stimulate an open discussion of topics and issues that were most salient for respondents rather than to impose the researcher's framework of understanding [25]. After the pharmacy observations, pharmacists and support-staff interviews were held to discuss their perceptions of MURs. Topic guides were developed to explore pharmacist (Box 2) and support-staff (Box 3) perceptions of the MUR service. As with the patient interviews, staff interviews were individually tailored to clarify, confirm and extend the observational data.

### 2.1 Analysis

Data analysis started during the early stages of data collection. The focus of the observations and the interview topic guide were revised during the data collection period. This inductive approach is considered to be good qualitative practice [25,26]. All observation field note documents were typed up and all the interviews transcribed verbatim. The data were then imported into N-Vivo8.

A thematic approach to analysing the qualitative data was used and involved initially reading and re-reading each section of the text and collating them under different headings or ‘codes’. The aim of coding is to ensure all the data that relates under the same heading can be retrieved with ease [26]. Categories from the interview transcripts and observation field notes were constructed based upon what was observed and reported in interviews. Box 4 provides an illustration of a section of the coding framework that was developed for the code ‘workload pressures and targets’. Coded extracts were then systematically read through and the contents condensed so that all the different issues that were raised were recorded. Consideration was then given to how these issues might be grouped together in broader themes which were then synthesised and narrated. The principle of constant comparison were used to test and refine these themes. A detailed explanation of the data analysis process used is described by Ziebland and McPherson [26].

## 3 Results

### 3.1 Study sites

The multiple pharmacy was located in a relatively affluent town, on a busy high street. The healthcare counter was located at the rear of the shop alongside the dispensary. The area did not allow private discussions so people could be overheard when speaking to pharmacy staff. The consultation room, installed to provide the MUR service, was located nearby and seldom used for other purposes. The room was well lit and contained two chairs and a small table.

The independent pharmacy was in a similarly affluent but residential suburb. The dispensary was behind the healthcare counter towards the rear and raised above the rest of the shop. Again there was no obvious place for private discussion. The consultation room was next to the dispensary and had been adapted for MURs from an existing office. The room had a window with net curtains allowing privacy and was rarely used for discussions with patients other than MUR consultations.

#### 3.1.1 Pharmacist counseling during supplies of prescription medicines

Field notes included observations and reflections from 114 patient-staff dispensing interactions (see Box 5 for coding framework). Observations revealed that most patients came to the dispensary to fill their prescriptions. Patients’ initial interaction was usually with pharmacy support-staff and limited to enquiries about when the patients’ medicines would be ready to collect. Phrases typically used by the pharmacy staff to indicate their agenda to fill the prescription included ‘*Are you waiting or calling back*?’ or ‘*It’ll be 10 minutes. Do you have any shopping to do?*’ Pharmacists were heavily involved in the dispensing process and their interactions with patients were brief and primarily to hand out medicines. The information provided was often generic in nature:

*Rebecca [pharmacist]: This is a new item isn’t it?*

*Patient: Yes. [Mentions it is for his shoulder]*

*Rebecca: Yes, ok then. [Patient describes his shoulder pain. Rebecca, turning sideways provides a cue that she wants to return to the dispensary].*

*Rebecca: It can cause drowsiness.*

*Patient: That's what the doctor said. Is there enough to last me for a fortnight?*

*Rebecca: If you take less than 8 a day, then yes. Bye.*

Observation Wk2 Independent

The above extract is a representative illustration of the unilateral approach that pharmacists took to counseling [19]. In this case, information about drowsiness was transmitted without first establishing whether the patient understood or was knowledgeable about this. During routine collection of prescriptions, information was typically delivered in an instructional manner with little two-way communication as illustrated by the following representative extract:

*Jane [pharmacist]: There's a note on your prescription that your metformin has been reduced from three times a day to twice a day, is that right?*

*Patient: Yes.*

*Jane: Do you know about that?*

*Patient: Yes.*

*Jane: I wanted to make sure.*

Observation Wk3 Multiple

Patients appeared comfortable with, or at least to accept this arrangement, for the provision of information. Questions asked by the pharmacist such as ‘*have you had this before?*’, ‘*has the doctor gone through this with you?*’ or ‘*do you know your dose of [medicine] has been increased*?’ received minimal responses from patients. Others have argued that the scope for providing new advice to patients is limited [21]. Occasionally, patients’ asked questions, but these were mostly to clarify the practicalities of taking the medicine, such as the dose. There was an assumption that patients knew about their repeat medicines. However, evidence suggests that this may not be the case and additional support may be required [27,28] even though patients may believe that routine information on repeat medication is unnecessary and pharmacists are accessible for further information if required [29,30]. Pharmacists, working within real life constraints of a busy dispensary, were not observed actively identifying patients who may have needed additional support but rather confined their input to confirming the doctors’ instructions.

#### 3.1.2 Pharmacist–patient interactions over-the-counter

Field notes included observations and reflections on 100 customer-staff OTC interactions (see Box 5 for coding framework). The wide range of medicines and retail products available meant that there was an apparent freedom for customers to ask about a variety of health issues. OTC sales of medicines were routinely undertaken by medicines counter assistants (MCA). Customer–MCA interactions have been shown to be complex, characterised by multiple discourses in which both parties commit, to legitimise the MCA as a medical advisor [31]. Customer–pharmacist discussion in OTC interactions tended to be conversational and tailored to patients’ specific requests for advice and information:

*Customer: Which is better? [Holds up two athletes foot products]*

*Jane [Pharmacist]: Is the inside moist or dry?*

*Customer: It's dry.*

*Jane: It's best to go for the cream. If it was moist you could have used the powder to dry it up…*

*Customer: Do you want to look? [Shows foot]*

*Jane: It looks moist, so use the powder…and you can use it in the socks as well…*

*[Patient purchases the medicine]*

Observation Wk3 Multiple

As is illustrated in the above extract OTC discussions were more open and conversational in nature than counseling on prescribed medicines. In fielding enquiries directly from customers, the pharmacist often needed to establish something about the customers’ circumstances and an understanding of the problem before recommending a treatment. The interactional focus was more ‘patient-centred’ or, as previous literature suggests, consumer-led [21]:

*[A woman enquires about a new anti-obesity drug]*

*Customer: It goes straight through does it? [Yes]. I don’t want to use it then because I use cod liver oil in the morning …I don’t have to take it in the morning do I?*

*Rebecca [Pharmacist]: No. You can take it at lunchtime and in the evening.*

*[Discussion continues, after which patient purchases the medicine]*

Observation Wk5 Independent

In this illustrative extract the pharmacist discussed with the customer adjusting the dose of the medicine according to her needs. However, the pharmacist neglected to enquire into the customer's lifestyle or other matters that may have been relevant to her weight management. Nevertheless, many OTC interactions served to address specific customer-initiated requests for advice and resembled what Pilnick describes as a ‘stepwise’ counseling approach, where knowledge and competence were explored in the encounter [19]. In these circumstances, customers who approached the pharmacy staff for advice appeared both familiar with and accepted the role of the pharmacist as an accessible adviser. However, the shop-floor environment was not conducive to more detailed or personal discussions.

#### 3.1.3 MUR interactions

##### 3.1.3.1 Participant response rate

Fifty-four patients gave consent for AL to observe their MUR consultation [32] and 34 patients agreed to be interviewed about their experience of the MUR. Most patient interviews were conducted at the pharmacy (two at the University), lasted around 45 minutes and all were audio-recorded.

#### 3.1.4 The MUR consultation (see Box 5 for coding framework)

When inviting patients, pharmacists and staff often presented the MUR as a quick activity to “*check*” their medicines. Once patients had agreed and were seated, MUR consultations followed a similar pattern, directed by the pharmacist's use of the MUR form. Some patients reported surprise at being approached ad hoc. This was a deviation from the standard pharmacist interaction described above, and to which they were accustomed and so they felt unprepared for an extended consultation. However, most did not mind this approach [33]. Pharmacists began with a brief explanation of the purpose of the MUR. Patient–pharmacist roles and expectations were therefore constructed from the onset through the pharmacist's announcement of the agenda:

*Kate [Pharmacist]: This is to check your medicines that you’re on and that they’re not interacting with anything over-the-counter…*

MUR 30 – Multiple

A question–answer sequence then followed where the pharmacist would ask the patient questions about their prescribed and OTC medicines. This enabled the MUR form to be completed quickly. All pharmacists were observed simultaneously completing the MUR form and talking to patients. Patients typically offered minimal responses to the closed nature of the pharmacist's questions:

*Jane [Pharmacist]: The Feldene, how often do you use it? [Patient replies that she uses it when she gets arthritis].*

*Jane: So you know what it's for?*

*Patient: Yes.*

*Jane: You don’t get any irritation?*

*Patient: No.*

*Jane: The cetirizine, you know what that's for?*

*Patient: A rash.*

*Jane: Are you alright swallowing that?*

*[MUR continues]*

MUR 11 – Multiple

This extract illustrates the procedural way most MURs were carried out by this pharmacist. All pharmacists dominated the consultation with nearly half of all patients observed not to ask any questions during the encounter (Table 1). Pharmacists did allow patients to talk and responded adequately to queries such as whether medicines could be taken together. However, they relied heavily on reference to the prescriber's instructions to circumvent or close down discussion of more indeterminate medicine-related matters:

*Jane [Pharmacist]: The fleconide, you take two twice a day?*

*Patient: I take one twice a day.*

*Jane: The doctor's got you down as two twice a day.*

*Patient: I take one in the morning and one at night.*

*Jane: You need to have a word with the doctor…*

MUR 19 – Multiple

Although MURs aim to investigate patient's understanding and use of medicines, patients often spoke of their illness. In several MURs pharmacists were seen to be cautious about prying into patients’ medical affairs and in their interviews revealed an aversion to discussing sensitive issues. As is exemplified by the following extract the format of the MUR did not facilitate a discussion of the patient's medical condition or related concerns:

*Rose [Pharmacist]: The Gaviscon, do you take two spoons at night? [Pharmacist enquires why]*

*Patient: [yes] …for silent reflux. It occurs when tiny particles of acid make their way up the oesophagus, damaging the vocal chords…They don’t know if it's silent reflux or an ulcer*

*Rose: I’m going to put here ‘nerves not kicking in properly’. I don’t need to give you any more information, but you probably know that it forms a raft on the contents of your stomach and so it prevents the reflux… [Conversation turns to the next medicine].*

MUR 7 – Independent

The above extract shows a lack of pharmacist's curiosity to explore the uncertainty expressed by the patient about her diagnosis resulting in a lost opportunity to discuss something the patient might have found useful. Instead, the pharmacist provided information about how the medicine worked. Likewise, pharmacists were observed frequently embedding unsolicited advice about side effects on medicines in their discourse with patients. On a few occasions, patients did indicate during the MUR that they found the information provided useful:

*Patient: I have some antihistamines… that's ok isn’t it with the co-proxamol?*

*Rebecca [Pharmacist]: If they’re the one-a-day ones then they’re fine with everything that you’re on…if you have dry eyes, and you find that you are using the drops more, than it might be the antihistamines that are doing that.*

*Patient: That's worth knowing…*

MUR 13 – Independent

Patients accepted advice when the pharmacist was able to frame the perceived benefits for the patient or convince them of avoiding future harm:

*[Conversation turns to the patients’ knee pain]*

*Patient: He [GP] did tell me to take glucosamine…*

*Jane [Pharmacist]: …With condroitin, now you need to talk with the diabetic nurse about this as they have found that it can interfere with your diabetes…*

MUR 15 – Multiple

As a result of his MUR, the patient had stopped his glucosamine tablets and was going to speak to the diabetic nurse and his GP. Although the patient was content to do this, the pharmacist did not feel the need to contact the nurse or GP directly. Likewise, when patients revealed an unusual side effect or reported following personal regimes of medicine taking, the pharmacist dealt with these issues in a succinct and apparently superficial manner:

*Patient: …I find sometimes at night if I take three paracetamol for the pain it works.*

*Rebecca [Pharmacist]: You shouldn’t really do that.*

*Patient: I find if I take three when the pain is bad it gets me to sleep.*

*Rebecca: Well, it's best to take two.*

*Patient: I don’t do it often…*

MUR 18 – Independent

As the extracts illustrate, the response of the pharmacist allowed the MUR to continue. However, the patient revealed in her interview a lack of acceptance of the pharmacist's advice as she reported that she would persist in her habit of taking three paracetamol. Rather than working with the patient, pharmacists took an inflexible view in circumstances where medication was being used in ways other than had been prescribed. The questions asked during the MUR consultation were therefore not focused on how to manage the patient's illness better with the aid of medicines, but whether the medicines were being used in a way that was acceptable in terms of the pharmacist's opinion and advice. Pharmacists occasionally asked at the end of the MUR whether the patient had any questions about their medicines; few responded to take up the invitation.

#### 3.1.5 Patient experiences

Patients’ reported being unfamiliar with the MUR, supporting previous research indicating patients’ low expectation and knowledge of the pharmacist's role [34]. Thirteen of the 37 patients interviewed reported previously having had an MUR; however, few could remember any details of this or actions taken as a result. The lack of purpose for the MUR, patients’ inability to set their own agenda, as well as the structured way the consultation was framed, left some patients with the impression they were helping with a task the pharmacist was required to complete rather than taking part in a consultation for their own benefit:

*Esther: I don’t mind, to me if I can help...I like to do it and it makes me feel good that I’ve helped somebody…*

Patient interview – Independent

Most patients reported their MUR had not improved their knowledge and rarely affected their use of medicines (Box 6). Many patients already felt knowledgeable about medicines that had been prescribed for their long-term conditions. Nevertheless, a few patients reported parts of their MUR to be useful:

*Mia: …she said you need to go and see the asthma nurse…I thought I was on the most I could go on and I’d have to tolerate it but she said that they can help you more…*

Patient interview – Multiple

*Comfort: I think it gives you more confidence, it does me, gives me more confidence to think I’m doing the right thing and taking the right medicine*

Patient interview – Multiple

Most patients appreciated and valued the time the pharmacist spent with them and described the MUR as “*satisfying*” or “*interesting*”. The pharmacist was viewed as a knowledgeable expert and this served to reassure patients about their medicines (Box 6).

#### 3.1.6 Staff views

##### 3.1.6.1 Participant response rate

All pharmacy staff within both pharmacies were invited to be interviewed to discuss their perceptions of MURs. Five pharmacists who had been observed throughout the study were interviewed as well as 12 (out of 14) regular support-staff.

#### 3.1.7 Organisational constraints

Every one of the five pharmacists was observed adopting several roles, as evidenced by other research [34], including dispenser, patient advocate, manager and health service provider. Pharmacists’ heavy engagement in the dispensing process meant that most MURs were performed opportunistically when the pharmacy was less busy. With no additional staffing, MURs had been poorly integrated into the pharmacists’ daily activities. In their interviews, pharmacists indicated that they felt constrained to speed through the MUR by the need to return to their ‘routine’ duties in the pharmacy (Box 6). Pharmacists, particularly those working for the multiple, reported feeling pressurised to deliver a targeted number of MURs. One pharmacist revealed that she purposefully chose patients on fewer medicines or simpler regimes that could be performed quickly despite acknowledging that those on more medicines or complex regimes potentially stood to benefit more:

*Jane: … you see a massive script, you think I don’t want to do an MUR on that. But though, probably they would be the best people who would get the most out of it. You see a prescription that's got maybe two items on it, dead easy…the emphasis is on targets so it's quantity and not necessarily quality…So I think people are trying to get, do the easiest ones possible to get the numbers rather than concentrating on getting those that perhaps would benefit from it.*

Employee pharmacist, Multiple

Support-staff reported tensions with patients who were awaiting prescriptions and with those wishing to speak with a pharmacist while the pharmacist was performing an MUR (Box 6). Support-staff were aware that they could interrupt the pharmacist in these circumstances and made personal judgements between interrupting a private consultation and appeasing waiting patients.

## 4 Discussion and conclusion

### 4.1 Discussion

Pharmacists in this study adhered to a format for conducting MURs which was largely determined by the structure of the MUR form. Although pharmacists fulfilled their obligation of asking the questions, the consultation left patients little scope for a more open discussion of their medicines. Pharmacists delivered information in an instructional manner in a way that was similar to their interaction with patients while dispensing medicines. These findings support previous literature indicating that the pharmacist's conversational turn aims to promote their agenda rather than altering in response to what the patient said; the pharmacist remained focused on the medicine rather than the patient's illness [35,36]. MURs have been criticised for promoting a professional agenda focusing on patient compliance, rather than concordance [37]. Consultation skills such as responding to patient cues, using open questions and eliciting the patient's perspective have been identified by others as areas that pharmacists need to improve [38]. Constrained by their remit, pharmacists were reluctant to engage with contentious or problematic issues and this meant that opportunities were lost for meaningful engagement. Far from providing an opportunity to develop skills and extend their professional authority, the routinised nature of the MUR reduced the pharmacists’ scope for exercising professional judgement and autonomy. Nevertheless, most patients valued the time the pharmacist spent with them and the privacy of the MUR consultation. They saw pharmacists as knowledgeable experts who were able to reassure them about their medicines, supporting evidence that suggests benefit in developing community pharmacists’ role both as a reviewer of patients’ medicines and as an adviser [39].

In contrast to their handling of the MURs, this study showed that pharmacists could provide customised information in responding to patient requests for advice about minor ailments. In these circumstances, patients appeared both familiar with and accepted the role of the pharmacist as an accessible adviser. The autonomy and willingness of the pharmacists to accommodate patient preferences during OTC discussions was in contrast to their constrained approach when discussing prescribed medicines during the MUR. Whereas the pharmacists’ role in dispensing and OTC activity could be conceptualised as a transformative activity, where the inert drug is transformed into a medicine for the individual patient [40,41], MUR activity was not observed to contribute to this process. Adopting a formulaic approach when filling out the questions on the MUR form left little room for discussions beyond the simple ‘checking’ of medicines.

The perceived pressure to pursue a targeted number of MURs was more prominent within the multiple and pharmacists reported dissatisfaction over the issue. Other studies have reported similar findings [42,43] suggesting that pharmacies are being remunerated for MURs which are not targeted at patients who may benefit most, leading to concerns over their value [44]. In this way, the commercialisation of MUR activity challenged rather than enhanced pharmacists’ professional status; a finding highlighted by others [42,45]. Harding and Taylor warned simply: ‘*asking structured, formulaic questions*’ [41]. The MURs observed were at odds with policy commitments and strategies to develop a responsive service which is individually tailored and patient-centred [1,9].

To our knowledge this is the only observational study that has explored patient–pharmacist interactions and MUR consultations as they occurred naturally in practice. These findings make a significant contribution to the analysis of current pharmacy practice adding to existing doubts about the extent to which the MUR service is meeting its stated aims [42]. Limitations of this study include the unknown effect of the researcher's presence on the pharmacy staff's behaviour and on the pharmacist and patient during the MUR consultation. The longitudinal nature of the study was intended to reduce the extent to which participants modify behaviour as a result of a heightened awareness of the observer. Furthermore, audio or video recording the MUR consultation would have provided verbatim data. Nevertheless, it was decided that hand written notes would be used by the researcher to record the MUR consultation because of ethical considerations [32]. Only two pharmacies were investigated and the observations made by one researcher. Further research of patients’ experience of MURs is clearly needed and in a wider and more diverse range of community pharmacy settings. Additional research should also investigate whether better targeting of MURs improves their outcome or whether this depends upon widening the pharmacist's remit when performing an MUR and improving their consultation skills.

### 4.2 Conclusion

MURs provide a nationally recognised counseling role for UK community pharmacists. However, several factors hindered pharmacists’ capacity to engage effectively with patients. Pharmacists’ heavy commitment to the dispensing process meant there was poor integration of the MUR service into their routine workload. Patients were unaccustomed to having a consultation with the pharmacist and lacked awareness of what it could offer. Consultations therefore served professional objectives rather than being patient-centred. The interactions and advice given within MUR consultations were predominantly instructional and resembled interactions about dispensed medicines. During OTC consultations, pharmacists involved patients to a greater extent indicating they already have many skills to effectively engage patients in decisions about their healthcare. However, pharmacists failed to take full advantage of the opportunities offered by MURs to fully involve patients, being constrained by situational pressures and commercial intent.

### 4.3 Practice implications

Although well intentioned, the MUR service is not realising its aim to improve patients’ knowledge, understanding and use of medicines. Substantial changes to current practice are needed. If pharmacists are to engage in new consultative services a review of their communication skills training is required so they can better elicit and engage with patients’ perspectives and concerns about taking medicines. Changes to policy also need to be considered particularly the way organisations implement and incentivise pharmacists to perform MURs so that patients who may benefit most are better targeted and pharmacists have the requisite space and time to incorporate MURs within their routine practice.

## Conflict of interest

AL was an MUR accreditor for De Montfort University, Leicester, UK.

## Role of the funding source

AL's PhD was funded by the Medical Research Council and Economic and Social Research Council. The views expressed are those of the authors.

## Figures and Tables

**Table 1:** Demographic data and characteristics of MUR consultations (*n* = 54).

Outcome measure	Independent pharmacy	Multiple pharmacy
Number of MURs observed	21	33
Patient gender		
Men	7	8
Women	14	25
Mean age of patients (range)	65 (46–81)	72 (40–89)
Number of patients invited by the pharmacy staff ad hoc or via appointment		
Ad hoc	17	31
Appointment	4	2
Mean number of medicines per patient^a^ (range)	8 (2–17)	6 (2–11)
Mean number of questions asked by the pharmacist during the MUR^b^	10	14
Number of patients who did not ask any questions during the MUR	12 (57%)	13 (39%)
Of the remaining patients, mean number of questions asked per patients (range)	2 (1–4)	3 (1–9^c^)
Number of patients who previously had an MUR (revealed in the patient interview)	5 in 17 interviews	7 in 17 interviews

a This was the number of prescribed and OTC medicines that were mentioned by the pharmacist or patient during the MUR and interviews.

b The number of direct questions asked by pharmacists is presented here. However, pharmacists also used statements to confirm that patients were taking a particular medicine or taking a particular dose. These have not been included in the count.

c There was one MUR where a patient and a carer were both present. Nine questions were asked; the carer asked five questions (one about his own health) and the patient asked four questions.

## Box 1 Patient interview topic guide

Demographic details

About the MUR:

1. Opening question: could you tell me from beginning to the end your experience of the MUR in as much detail as possible?
2. Respondent's awareness of MUR service & views of being approached.
3. Respondent's expectations & views of purpose of the MUR.
4. Exploration of what happened during the MUR (using observation notes).
5. Views on necessity/usefulness/would respondent like to have discussed anything?
6. Affect knowledge or use of medicines?
7. Likes and dislikes about review?
8. Who in your opinion would most benefit from MUR?
9. Improving the service/another MUR in future?

Pharmacy use & perceptions around role of the pharmacist.

Respondent's medicines & medical care.

1. Beliefs, necessity and concerns over medicines.
2. Perceived authority over medicines.
3. GP & other health professional role in respondent's care.

## Box 2 Pharmacist interview topic guide

Demographic data & accreditation process.

Pharmacist's experience of MURs:

Patient selection.
Views & use of MUR forms.
Necessity of MURs.
Most common concerns patients have about their medicines?
Organisational pressure & targets.
Professional boundaries.

Objectives of MUR:

What do you hope to achieve? Good outcome/bad outcome.
View on improving knowledge and use of patients’ medicines.
Resolving patient's ineffective use of medicines & examples.
How often do you make suggestions/are these accepted?
Views on value for money for NHS.

Support:

Do you welcome MURs/has this added anything to your role?
What support have you had to help you develop the service? (employers, local surgery, Primary Care Trust)
How can MURs be improved?

## Box 3 Pharmacy support-staff interview topic guide

Demographic data.

Training received.

Patient selection.

Identifying patients.
Explore patient responses.

Organisational issues:

View on pharmacist performing MURs.
Managing work without pharmacist.
Examples where the pharmacist was needed but was unavailable & patient response.
Organisational pressures & MUR targets.

## Box 4 Illustrative section from the coding framework. A selection of sub-codes is provided with an example of associated text for the main code ‘workload pressures and targets’

Workload pressures and targets:

 Workload pressure *… the dispensing bench had several piles of prescriptions…to be checked by the pharmacists. Jane [pharmacist] had decided that the workload was too much…she told Kate [pharmacist] that ‘we need to catch up so just book MUR appointments’…there was no negotiation…Subsequently no MURs were performed that afternoon.* * Extract from observation notes. Multiple pharmacy.*

 Patient views on pharmacist performing MURs: *Beth: I know they’re very busy people these pharmacists aren’t they… they’re always rushed off their feet…I don’t think they should be pulled from there to do that sort of work, in that room, reviews and then have to go back again…sometimes that's how mistakes are made, they’re rushed…* Patient interview, Multiple

 MUR targets: *Rebecca: Well, she kind of tries to set a target, but does understand when it's not met…she does say “try and do one MUR a day” but then, realistically, when she looks at the figures she's not sort of on your case…* Employee pharmacist interview, Independent

 Support-staff views on targets: *Dawn:…Nothing should be causing pressure like that when you’re dealing with medication…You shouldn’t have targets…It should be as and when a customer, you feel, needs a medication review. It shouldn’t be about, you’ve got to do ten today. Because if you do ten and you don’t give them the quality of service, what's the point in it...?* Support-staff (dispenser) interview, Multiple.

## Box 5 Coding framework for OTC, dispensing and MUR counseling interactions

OTC:

 Accessible advisor

  Patient expectations/establishing need

  Resolver of problems

 Patient ambiguity of role

 Commercial environment & influence on counseling

 Range of OTC counseling

  Emergency hormonal contraception consultations & confidentiality

  Nicotine replacement therapy consultations

  Other OTC pharmacy activities

 Indeterminacy in OTC work

 Lack of information during interactions

 Organisational culture

 Power struggle patient/pharmacist/GP

 Privacy

 Rapport with patients

 Support-staff referrals

 Work flow management

Dispensing:

 Double checking with patients

  Role as checker

 GP issues & influence

  Law bound

  Subordinate to GP

 Environment – not facilitative for counseling

 Lack of information

 Range of dispensing counseling

  Medidose

  Methadone

  Prescription

 Paternalism

 Points out changes

 Pharmacist holds specialist knowledge

 Pharmacist activities viewed by patients

 Pharmacist as controller of medicines

 Pharmacist definitive source of information

 Pharmacist sociable

 Pharmacist natural attitude

 Resolver of practical medication issues

 Refers to GP when required

 Resolver of dispensing errors

 Trust

 Will NOT explore issues further

MUR:

 Space issues in consultation room

 Initiation of MUR

  Lack of patient focus

  Appears rushed

 Confusing or inappropriate questions (pharmacist)

 What is happening in MUR

  Checking medication

  Expert patient

  Missed interactions

  Problems establishing use

  Educating patients

   Information giving

   Informing about changes

   Resolving side effects

   Patient confusion about MUR

   Patient education about MURs

   MURs as big brother

  Explaining medication use

   Gray or indeterminate area

   Identifying potential future problems

   Interpersonal relationship

   Lacks patient focus

   Other issues not medicine linked

   Patient asks about side effects

   Patient happy with response

   Problem with medications

 Prescriptive nature of MUR

 Problems of establishing patient use of medication

 Pharmacist does not know answer

 Patient aversion to medicines

 Superficial questions/lack probing

 Time pressures

## Box 6 Representative quotations from patient and staff interviews

Patient interviews

Theme: awareness and invitation: *Researcher: Do you remember how you were approached initially?* *Primrose: The lady came up to me and said would I mind going through my medication with the pharmacist and just to kind of make sure that we both knew why this medication was being prescribed and it was just something that chemists are having to do now.* Patient interview – Independent *Researcher: Were you expecting a review?* *Nick: No [laugh] no, Jane [pharmacist] just collared me [laughter].* Patient interview – Multiple

Theme: knowledge and use of medicines: *Eve: The only thing I was more knowledgeable about was when she told me it was all right to take them like that [to take tablets together]…it put my mind at rest…* Patient interview – Independent. *Jill: [Sighs] Well I don’t think I’ve got no more knowledge, I think it's just that I’ve been on these for so long, and once you’ve been on them for so long the doctor does makes sure that you’re alright with them.* Patient interview – Multiple

Pharmacist interviews

Theme: workload issues: *Linda: …I’m quite keen on them [MURs] but it's a time restraint and don’t feel like I want to rush people through doing one, but I don’t want to feel stressed that I’ve got staff out in the shop getting stressed because the customers are getting stressed because you’re not there and what have you...* Locum pharmacist, Independent *Researcher: …how conscious are you that you’re going to be returning to a full checking bench?* *Jane: Very. You can’t switch off from the fact that you know that's going on. And that's in the back of your mind…when you know that you’re exceptionally busy, it puts you under a pressure and you really just want to get through that, those questions as quickly as possible. Because, you know you’re going to go back to bedlam…You can’t switch off from that, because that's part of your responsibility as well.* Employee pharmacist, Multiple

Theme: MUR target & pressure: *Rebecca: …And I think Rose [owner] definitely understands though, which is why she has totally said that, I know it's very difficult to go from a busy setting, like you say, in the checking mode, in that mode and then to go to this whole consultative role…* Employee pharmacist, Independent *Researcher: …what support have you had with these Medicines Use Reviews? Apart from the support to be accredited?* *Kate: Yeah, no, they just pretty much throw you in at the deep end, they just don’t want to know. They just want to know if you’re meeting target…* Employee pharmacist, Multiple

Support-staff interviews

Theme: Absent pharmacist: *Lucy: I think it's frustrating from the fact that you know people are waiting for prescriptions and you’re having to say ‘the pharmacist has got a patient in with her at the moment’. People just want to go, don’t they? They want their prescription, they’re not bothered about why she's interviewing another patient…* Dispenser, Independent *Leah: There's also people that come in and ask specifically to speak to the pharmacist … they don’t want us, they want the pharmacist…* Counter assistant, Multiple

Theme: Staffing: *Cath: …it's not practical for us to get two pharmacists …it's expense you’re paying for two pharmacists at once… again it's money isn’t it? How practical would it be to get another pharmacist…* Counter assistant, Independent *Stef: …they won’t give us two pharmacists every day will they…so long as one is available.* Counter assistant, Multiple
